# The impact of image and performance enhancing drugs on atrial structure and function in resistance trained individuals

**DOI:** 10.1186/s44156-023-00031-y

**Published:** 2023-12-06

**Authors:** Florence Place, Harry Carpenter, Barbara N. Morrison, Neil Chester, Robert Cooper, Ben N. Stansfield, Keith P. George, David Oxborough

**Affiliations:** 1 Research Institute for Sport and Exercise Sciences, Liverpool John Moores University, Tom Reilly Building, Byrom Street, Liverpool, L3 3AF UK; 2 School of Human Kinetics, Trinity Western University, Langley, BC Canada; 3 Department of Pharmacology and Toxicology, College of Pharmacy, University of Arizona, Arizona, USA

**Keywords:** Performance-enhancing drugs, Resistance training, Echocardiography, Speckle tracking, Strain

## Abstract

**Background:**

Image and performance enhancing drugs (IPEDs) are commonly used in resistance trained (RT) individuals and negatively impact left ventricular (LV) structure and function. Few studies have investigated the impact of IPEDs on atrial structure and function with no previous studies investigating bi-atrial strain. Additionally, the impact of current use vs. past use of IPEDs is unclear.

**Methods:**

Utilising a cross-sectional design, male (n = 81) and female (n = 15) RT individuals were grouped based on IPED user status: current (n = 57), past (n = 19) and non-users (n = 20). Participants completed IPED questionnaires, anthropometrical measurements, electrocardiography, and transthoracic echocardiography with strain imaging. Structural cardiac data was allometrically scaled to body surface area (BSA) according to laws of geometric similarity.

**Results:**

Body mass and BSA were greater in current users than past and non-users of IPEDs (*p* < 0.01). Absolute left atrial (LA) volume (60 ± 17 vs 46 ± 12, *p* = 0.001) and right atrial (RA) area (19 ± 4 vs 15 ± 3, *p* < 0.001) were greater in current users than non-users but this difference was lost following scaling (*p* > 0.05). Left atrial reservoir (*p* = 0.008, *p* < 0.001) and conduit (*p* < 0.001, *p* < 0.001) strain were lower in current users than past and non-users (conduit: current = 22 ± 6, past = 29 ± 9 and non-users = 31 ± 7 and reservoir: current = 33 ± 8, past = 39 ± 8, non-users = 42 ± 8). Right atrial reservoir (*p* = 0.015) and conduit (*p* = 0.007) strain were lower in current than non-users (conduit: current = 25 ± 8, non-users = 33 ± 10 and reservoir: current = 36 ± 10, non-users = 44 ± 13). Current users showed reduced LV diastolic function (A wave: *p* = 0.022, *p* = 0.049 and E/A ratio: *p* = 0.039, *p* < 0.001) and higher LA stiffness (*p* = 0.001, *p* < 0.001) than past and non-users (A wave: current = 0.54 ± 0.1, past = 0.46 ± 0.1, non-users = 0.47 ± 0.09 and E/A ratio: current = 1.5 ± 0.5, past = 1.8 ± 0.4, non-users = 1.9 ± 0.4, LA stiffness: current = 0.21 ± 0.7, past = 0.15 ± 0.04, non-users = 0.15 ± 0.07).

**Conclusion:**

Resistance trained individuals using IPEDs have bi-atrial enlargement that normalises with allometric scaling, suggesting that increased size is, in part, associated with increased body size. The lower LA and RA reservoir and conduit strain and greater absolute bi-atrial structural parameters in current than non-users of IPEDs suggests pathological adaptation with IPED use, although the similarity in these parameters between past and non-users suggests reversibility of pathological changes with withdrawal.

## Background

Image and performance enhancing drugs (IPEDs) are used by resistance trained (RT) individuals to increase muscle mass, reduce body fat and improve strength and perceived body image [1]. These include; anabolic steroids, human growth hormone, clenbuterol and diuretics [2], among others. Over 1 million people in the UK are using IPEDs, with this value increasing each year [3], likely due to their wider availability in the recreational gym population [4]. IPEDs have harmful effects including cardiomyopathy, hypertension, hepatotoxicity and tendon rupture [5, 6]. Previous studies have demonstrated the cardiotoxic effects of IPEDs, including left ventricular (LV) hypertrophy [7, 8], with a similar presentation to hypertrophic cardiomyopathy [1], reduced LV function [8–10], myocardial fibrosis [11, 12] and increased coronary plaque volume [13, 14]. The outlined evidence, alongside a report highlighting that 75% of male gym users have considered IPED use [15] and recent sudden deaths of high-profile bodybuilders, emphasises the importance of understanding the cardiac effects of these substances.

Despite the impact of IPEDs on LV structure and function, few studies have investigated the impact on atrial remodelling. This focus is important because the atria act as a reservoir during ventricular systole and aids filling during diastole. The left atrium (LA) contributes to approximately 30% of cardiac output, strongly influencing overall cardiac function through the reservoir, conduit and booster phases [16]. Previously, atrial assessment has been limited to echocardiographic assessment of structural parameters, such as diameter, area, and volume. Speckle tracking echocardiography (STE) is used to assess myocardial strain, and is frequently being applied to the assessment of atrial function allowing detection of subtle abnormalities [17]. Left atrial strain has been demonstrated to be a surrogate of LV end diastolic pressure [18] and LA pressure [19] and may be more sensitive than conventional parameters including E/E’, E/A and LA volume index as well as providing an index of stiffness [20]. Additionally, a reduction in LA strain (conduit and reservoir) may occur prior to changes in parameters currently used to diagnose diastolic dysfunction i.e. lateral and septal E’ [21]. Similarly, right atrial (RA) strain rate has been correlated with RA pressure [22] and reservoir and conduit strain can be used to detect changes in right ventricular (RV) function, prior to changes in RV ejection fraction (EF) [23].

Of the few studies that have attempted to establish the association between LA structure and function in IPED users, a recent study of 35 male RT athletes (users n = 20, non-users n = 15) found impaired LA reservoir function in IPED users compared to non-users [24]. Similarly, Alizade [25] found that both decreased LA reservoir and conduit strain was associated with IPED use. Notably, both these studies, had a relatively small sample size, did not assess RA structure and function, and did not scale all chamber dimensions according to allometric rules. The importance of allometric scaling of cardiac dimensions within athletic populations has been previously outlined [26] and has greater importance in RT athletes, particularly IPED users, whereby muscle mass is increased [1]. Further, no previous studies have investigated atrial strain in past users. It has been shown that, following a period of IPED discontinuation, LV systolic function improved, whereas diastolic dysfunction remained impaired [13] and therefore atrial function may provide significant insight in this population.

In view of this, the aim of this study was to investigate bi-atrial structure and function in RT individuals. This overarching aim leads to two objectives, 1: to determine the impact of IPED use on atrial structure and function in RT individuals, and 2: to establish differences between past, current, and non-users. It is hypothesised that, 1: there will be a stepwise increase in absolute and allometrically scaled LA and RA size and volume from non to current users, and 2: current users will have the lowest reservoir and conduit strain, and lowest function with non-users exhibiting the best function.

## Methods

### Study population and design

Male (n = 81) and female (n = 15) RT individuals (age 29 ± 5 years) with a training duration of > 2 years and currently resistance training > 3 h per week were recruited into the study. Participants were grouped based on their self-reported IPED use status: current user defined as using IPEDs within 12 months of data collection (n = 57), past user defined as a previous user of IPEDs > 12 months from data collection (n = 19) and non-users defined as never using IPEDs (n = 20). Participants were excluded if they had a history of cardiovascular disease, diabetes, renal or liver disease, were pregnant or were over 80 years old. Participants provided written informed consent prior to participation. Ethics approval was obtained from the ethics committee of Liverpool John Moores University (reference 21/SPS/078).

The study utilised a cross-sectional design whereby participants were required to attend the laboratory on one occasion. Athletes completed a detailed questionnaire to capture any cardiovascular symptoms, family history and to determine specific IPED use. A 12-lead electrocardiogram (ECG) and comprehensive transthoracic echocardiogram were undertaken, and results were reported by a sports cardiologist with clinical referrals made if required.

### Procedures

#### Participant history

A training history was collected for all participants, including duration of training, training frequency (sessions/week) and training hours per week. A history of IPED use was collected from those in the current user group including names of substances, dosage, administration method, cycling history, duration of use and frequency of use. Those taking IPEDs were interviewed by a study investigator who had extensive knowledge in IPEDs to ensure details of IPED use were reported accurately and thoroughly.

#### Anthropometry and examination

Anthropometric testing included an assessment of standing height (Seca Supra 719, Hannover, Germany), body mass (Seca217, Hannover, Germany) and manual blood pressure. Body surface area (BSA) was subsequently calculated using the Mosteller equation [27].

#### Electrocardiography

A resting, supine 12-lead ECG was conducted according to Society for Cardiological Science & Technology (SCST) guidelines [28] using a commercially available system (Seca CardioPad-2, Birmingham, UK) and was interpreted in accordance with the International Criteria for ECG Interpretation in Athletes [29]. Heart rate was recorded.

#### Echocardiography

Transthoracic echocardiography was performed by a single British Society of Echocardiography (BSE) accredited sonographer using a commercially available ultrasound system (Vivid E95, GE Healthcare, Horten, Norway) with a 1.5–4 MHz phased array transducer, with the participant lying in the left lateral decubitus position. Images were obtained according to BSE minimum dataset [30] and athletic screening [31] guidelines. Images were stored as a raw digital imaging and communications in medicine (DICOM) format and exported to an offline analysis system (EchoPac version 202, GE Healthcare, Horton, Norway) for subsequent analysis.

#### Conventional parameters

An assessment of LV diastolic function included pulsed wave Doppler of transmitral flow during early (E) and late (A) diastole and E/A ratio was calculated. Mitral annular early diastolic (E’) and late diastolic (A’) velocity was measured in the apical four chamber (A4C) view for the septum and lateral walls and average E’, A’ and E/E’ were calculated. The parasternal long axis view was used to assess LA anterior–posterior dimension at end-systole. Apical two chamber (A2C) and A4C views were optimised to maximise LA length and volume at end-systole and LA volume (end-systole) was measured using Simpson’s biplane method. RA area was measured in the RV focused A4C view at end-systole. Atrial dimensions were presented as absolute values and also scaled allometrically to account for geometric similarity i.e. volumes indexed to BSA^1.5^ and linear dimensions to BSA^0.5^ [32].

#### Two-dimensional myocardial speckle tracking echocardiography

Speckle tracking echocardiography derived atrial longitudinal strain was obtained from A2C and A4C views for the LA and the A4C view for the RA. Images were optimised to ensure optimal atrial spatial resolution with frame rates between 40 and 90 FPS.

**Fig. 1:**
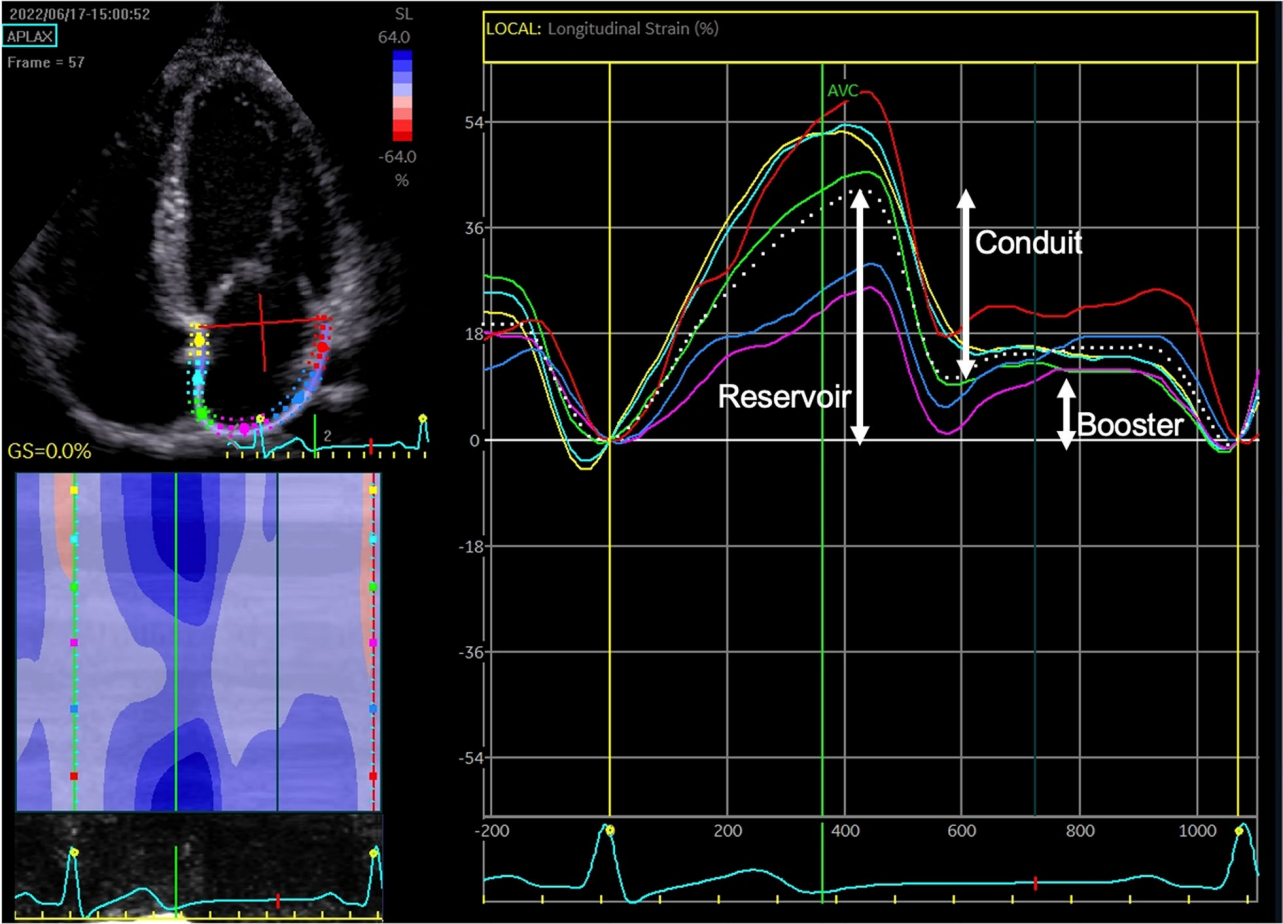
Measurement of left atrial reservoir, conduit and booster strain in the apical four chamber view

Offline analysis of strain was made in accordance with current guidelines [33]. A single cardiac cycle was used, and zero strain reference was set at ventricular end diastole. The region of interest (LA or RA myocardium) was traced and was divided into six equidistant segments which were then tracked, and global atrial strain for reservoir, conduit, and booster strain were calculated as an average of the six segments (see Fig. 1). Satisfactory tracking was determined by the software and validated by the operator. Left atrial stiffness index was calculated as the ratio of E/E’ to LA reservoir strain [20].

### Statistical analysis

Statistical analysis was performed using commercially available software package SPSS Version 28.0 for Windows (SPSS, Illinois, USA). All indices were assessed for normal distribution using a Kolmogorov–Smirnov test. Mean differences between current, past, and non-users were analysed using a one-way ANOVA with post-hoc Bonferroni adjustment for assessment of between group differences or the Kruskal–Wallis one-way analysis of variance for non-normally distributed indices. Associations between IPED use and atrial parameters were assessed using Pearson Product Moment correlation. Statistical significance was accepted at *p* < 0.05.

## Results

### Participant demographics and IPED use

The types of IPEDs used by current and past users are presented in Table 1. IPED users had a history of use of 6.8 ± 5.1 years. Median dose was 1108 mg week^−1^ [range (40–5400)] and users were taking 3.4 ± 1.6 [range (1–6)] substances simultaneously. Administration was primarily through injection (81% injection, 19% oral tablet).

**Table 1:** Image and performance enhancing drug use

	Current users (n = 57)	Past users (n = 19)	Administration method
Testosterone 400	31	5	Injection/Oral tablet
Test enanthate 300	45	6	Injection/Oral tablet
Human growth hormone	12	1	Injection
Boldenone	3	3	Injection/Oral tablet
Yohimbine	1	0	Not stated
Nandrolone decanoate	1	0	Not stated

Demographic and training data are shown in Table 2. Training load (duration and hours) were similar between groups [training duration (years): current = 12 ± 7, past = 12 ± 6, non-user = 11 ± 9 and training hours (hours per week): current = 11 ± 3, past = 9 ± 3, non-user = 9 ± 3; *p* > 0.05]. Weight (*p* = 0.002, *p* < 0.001), BSA (*p* = 0.004, *p* < 0.001) and heart rate (*p* = 0.003, *p* < 0.001) were greater in current users than past and non-users of IPEDs, respectively [weight (kg): current = 102 ± 18, past = 86 ± 16, non-user = 76 ± 14; BSA (m^2^): current = 2.24 ± 0.24, past = 2.02 ± 0.24, non-user = 1.89 ± 0.22; heart rate (bpm): current = 68 ± 11, past = 58 ± 11, non-user = 56 ± 9]. Systolic blood pressure was higher in current users than non-users (127 ± 10 mmHg vs 119 ± 11 mmHg, *p* = *0*.025), with no differences between past-users and any group (*p* > 0.05).

**Table 2:** Participant demographics

Variable	Current user Mean ± SD	Past User Mean ± SD	Non-user Mean ± SD
Sample size	57	19	20
Age (years)	30 ± 4^b^	27 ± 5	27 ± 6
Weight (kg)	102 ± 18^a,b^	86 ± 16	76 ± 14
Height (cm)	178 ± 9^b^	173 ± 11	170 ± 9
BSA (m^2^)	2.24 ± 0.24^a,b^	2.02 ± 0.24	1.89 ± 0.22
Heart rate (bpm)	68 ± 11^a,b^	58 ± 11	56 ± 9
Systolic blood pressure (mm Hg)	127 ± 10^b^	122 ± 13	119 ± 11
Diastolic blood pressure (mm HG)	74 ± 9	71 ± 8	72 ± 10
Training duration (years)	12 ± 7	12 ± 6	11 ± 9
Training hours (per week)	11 ± 3	9 ± 3	9 ± 3

^a^Denotes significance < 0.05 between current users and past-users

^b^Denotes significance < 0.05 between current users and non-users

### Atrial parameters

#### LA structural and functional parameters

**Fig. 2:**
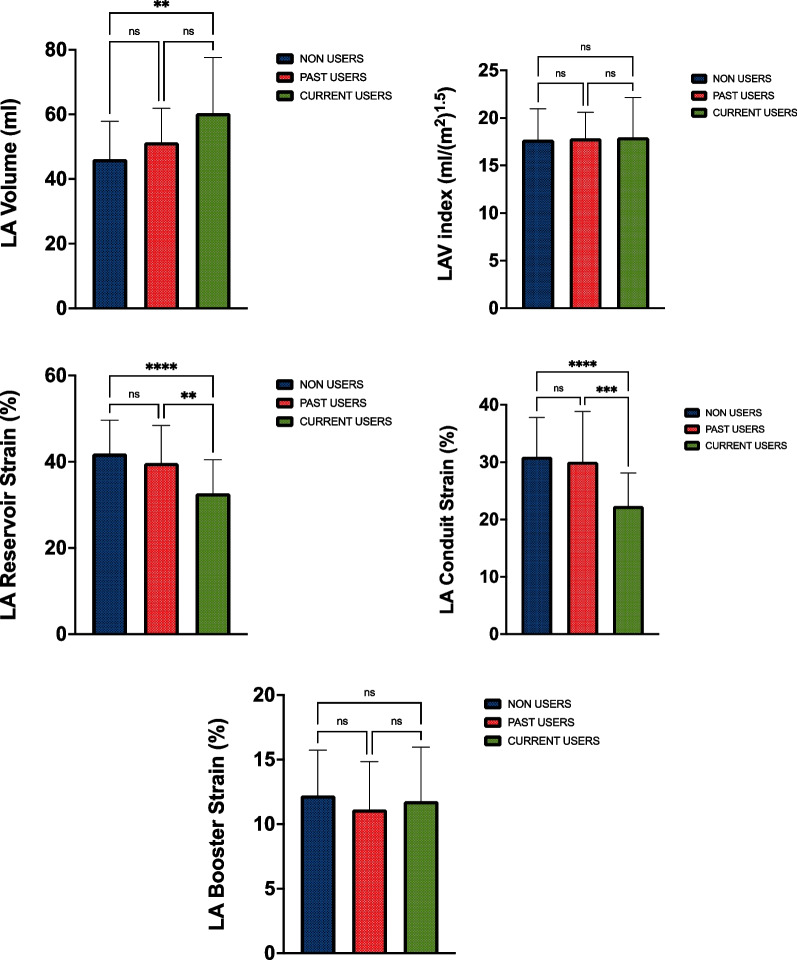
Left Atrial Structure and Function. *LA* left atria, *LAV* left atrial volume. *Denotes significance < 0.05. **Denotes significance < 0.01. ***Denotes significance < 0.001. ****Denotes significance < 0.0001

Figure 2 demonstrates LA structural and functional parameters. Absolute LA volume was greater in current than non-users (60 ± 17 ml vs 46 ± 12 ml, *p* = 0.001), however, there was no significant difference following allometric scaling (see Fig. 2 and Table 3). Past users showed no difference between either group in LA structural parameters (*p* > 0.05).

**Table 3:** Left atrial structure and function

Variable	Current user Mean ± SD	Past user Mean ± SD	Non-user Mean ± SD
LA anterior–posterior dimension (mm)	39 ± 5	38 ± 4	35 ± 4
LA anterior–POSTERIOR DIMENSION (indexed to BSA^0.5^)	26 ± 3	26 ± 2	25 ± 2
LA volume end systole (ml)	60 ± 17^b^	53 ± 10	46 ± 12
LA volume end systole (index to BSA^1.5^)	18 ± 4	18 ± 2	18 ± 3
LA reservoir strain (%)	33 ± 8^a,b^	39 ± 8	42 ± 8
LA conduit strain (%)	22 ± 6^a,b^	29 ± 9	31 ± 7
LA booster strain (%)	12 ± 4	11 ± 4	12 ± 4
LA stiffness	0.21 ± 0.7^a,b^	0.15 ± 0.04	0.15 ± 0.07

*LA* left atria, *BSA* body surface area

^a^Denotes significance < 0.05 between current users and past-users

^b^Denotes significance < 0.05 between current users and non-users

Left atrial reservoir (*p* = 0.008, *p* < 0.001) and conduit strain (*p* < 0.001, *p* < 0.001) were lower in current users compared to both past and non-users, respectively (conduit: current = 22 ± 6, past = 29 ± 9 and non-users = 31 ± 7 and reservoir: current = 33 ± 8, past = 39 ± 8, non-users = 42 ± 8). No significant differences were found in LA parameters between non-users and past users (*p* > 0.05). Left atrial conduit strain was significantly correlated with dose (mg wk^−1^) (*r* = 0.355, *p* = 0.025).

#### RA structural and functional parameters

**Fig. 3:**
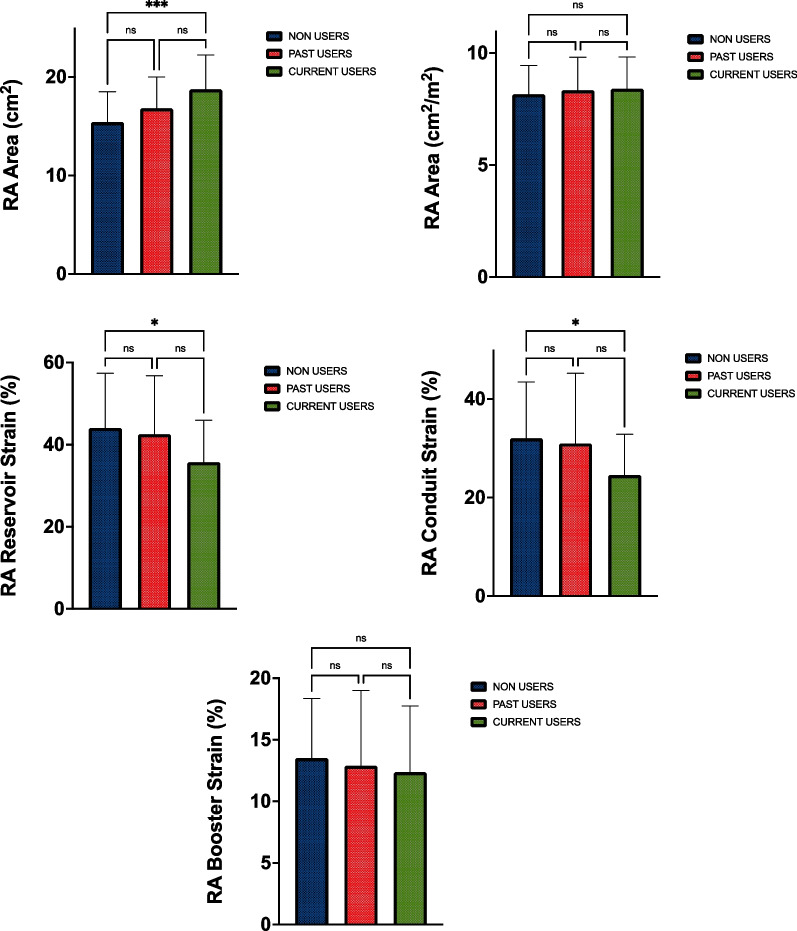
Right atrial structure and function. *RA* right atria. *Denotes significance < 0.05. **Denotes significance < 0.01. ***Denotes significance < 0.001

RA parameters are demonstrated in Fig. 3. Current users had a larger RA area than non-users (19 ± 4 cm^2^ vs 15 ± 3 cm^2^, *p* < 0.001), however, there was no significant difference following allometric scaling (see Table 4). Past users showed no difference between either group in RA structural parameters (*p* > 0.05).

**Table 4:** Right atrial structure and function

Variable	Current user Mean ± SD	Past user Mean ± SD	Non-user Mean ± SD
RA Area (cm)	19 ± 4^a^	17 ± 3	15 ± 3
RA Area Index (cm^2^ m^−2^)	8 ± 1	8 ± 2	12 ± 18
RA reservoir strain (%)	36 ± 10^a^	43 ± 14	44 ± 13
RA conduit strain (%)	25 ± 8^a^	31 ± 14	33 ± 10
RA booster strain (%)	12 ± 5	13 ± 6	13 ± 5

*RA* right atria

^a^Denotes significance < 0.05 between current users and non-users

Right atrial reservoir strain (*p* = 0.015) and conduit strain (*p* = 0.007) were lower in current than non-users (conduit: current = 25 ± 8, non-users = 33 ± 10 and reservoir: current = 36 ± 10, non-users = 44 ± 13). Past users showed no difference between either group in RA strain parameters (*p* > 0.05). Right atrial reservoir (*r* = 0.331, *p* = 0.034) and booster (*r* = 0.359, *p* = 0.021) strain were significantly correlated with dose (mg wk^−1^). Right atrial reservoir strain was significantly negatively correlated with number of substances used (*r* = -0.316, *p* = 0.044).

### Left ventricular diastolic function

Current users showed lower A wave (*p* = 0.022, *p* = 0.049) and E/A ratios (*p* = 0.039, *p* < 0.001) than both past and non-users (A wave (m s^−1^): current = 0.54 ± 0.1, past = 0.46 ± 0.1, non-users = 0.47 ± 0.09 and E/A ratio: current = 1.5 ± 0.5, past = 1.8 ± 0.4, non-users = 1.9 ± 0.4), see Table 5. E wave (m s^−1^) was lower in current users than non-users (0.78 ± 0.2 vs 0.89 ± 0.2, *p* = 0.018) and average E/E’ was lower in past-users than current users (5.5 ± 0.9 vs 6.7 ± 1.7, *p* = 0.010). There was no difference in LV diastolic function between past and non-users (*p* > 0.05). Current users also demonstrated higher LA stiffness than both past (*p* = 0.001) and non-users (*p* < 0.001) (current = 0.21 ± 0.7, past = 0.15 ± 0.04, non-users = 0.15 ± 0.07). Left atrial stiffness was significantly correlated with number of substances used (*r* = 0.328, *p* = 0.039).

**Table 5:** Left ventricular diastolic function

Variable	Current user Mean ± SD	Past user Mean ± SD	Non-user Mean ± SD
E (m/s)	0.78 ± 0.2^b^	0.79 ± 0.1	0.89 ± 0.2
A (m/s)	0.54 ± 0.1^a,b^	0.46 ± 0.1	0.47 ± 0.09
E/A ratio	1.5 ± 0.5^a,b^	1.8 ± 0.4	1.9 ± 0.4
Average E/E’	6.7 ± 1.7^a^	5.5 ± 0.9	5.9 ± 1.5

^a^Denotes significance < 0.05 between current users and past-users

^b^Denotes significance < 0.05 between current users and non-users

## Discussion

To the best of our knowledge this is the first study to assess LA and RA structure and function in a resistance athlete cohort of current, past and non-users of IPEDs. The main findings of this study were that current users (1) demonstrated higher absolute LA volume and RA area than both past and non-users however, when scaled allometrically for BSA these differences were removed and, (2) had reduced values of both LA and RA reservoir and conduit strain, higher LA stiffness and decreased LV diastolic function. In addition, there were no significant differences between past and non-users in LA parameters highlighting the potential reversibility of these findings through cessation of IPED use.

The atria significantly contribute to overall cardiac function, modulating ventricular function by acting as a reservoir during systole and a pump during diastole, attributing to ~ 30% of cardiac output [16]. As hypothesised, the current study showed a greater atrial size in current users compared to non-users of IPEDs, however there were no differences following allometric scaling to BSA (a surrogate for fat-free mass (FFM) in athletic populations [34]). Fat-free mass represents metabolically active tissue with up to 99% of metabolism taking place in the body cell mass [35] and is significantly elevated in IPED users [8]. Cardiac output is directly related to metabolism through tissue demands for oxygen [36] and increases to meet the demands of the larger muscle mass. This occurs through increased stroke volume (SV), brought about by increased absolute chamber volumes. This was supported by Whalley et al. [34] who found that both LV end diastolic diameter and LV mass were independently predicated by FFM in athletes. Additionally, studies [37–39] have found normalisation of structural cardiac parameters with allometric scaling in athletic populations. In IPED users, Morrison et al. [8] found significant differences in LV end diastolic volume and LV diameter between current users and non-users, with no difference following allometric scaling. It is therefore feasible to suggest that the differences in unscaled atrial size are due to increased body mass of IPED users and may be indicative of physiological adaptation according to the laws of geometric similarity.

Increased atrial size can be indicative of pathology. In a meta-analysis of 68 studies (n = 50,720), Froehlich et al. [40] found that LA diameter had a significant association with major adverse cardiac events, stroke and thromboembolic events in patients without atrial fibrillation (AF). Atrial diameter > 4.0 cm was also significantly associated with incident AF, stroke and death. Additionally, in hypertrophic cardiomyopathy, LA diameter, volume and strain show good predictive value of new onset of AF [41–43] and LA diameter is used within the European Society of Cardiology (ESC) guidelines [44]. This has further relevance with AF being the most common arrythmia seen in masters athletes with a prevalence greater than the general population [45], possibly due to increased atrial fibrosis or conduction disturbances [46]. The impact of IPED use on AF prevalence is unknown, however it can be speculated that the elevated chamber size demonstrated here may put these athletes at greater risk of developing AF in the future.

Atrial dilatation in pathological populations can also relate to both atrial myopathy [47] and diastolic dysfunction [48]. In hypertrophic cardiomyopathy patients, bi-atrial myocyte hypertrophy and disarray have been suggested to cause dilatation and contribute to the increased AF prevalence in this population [49]. During ventricular diastole, the atria are exposed to ventricular filling pressures [48]. In cases of decreased diastolic function and elevated filling pressures, atrial pressures must increase to maintain adequate ventricular filling and thus cardiac output [50]. This pressure overload increases wall tension, triggering myocardial hypertrophy and fibrosis [51], resulting in atrial dilatation. Therefore, LA volume is reflective of diastolic dysfunction severity [50]. Considering the lower LV diastolic function in current users compared to non-users (decreased E wave, A wave, E/A ratio and tissue Doppler imaging) in this study, alongside multiple previous studies linking IPED use to diastolic dysfunction [9, 10, 13, 52, 53], it can be suggested that this mechanism, may in part, be responsible for the LA dilatation demonstrated here, although longitudinal studies should aim to assess this further.

The addition of atrial strain into the standard echocardiographic examination has proven useful in other settings, including heart failure [21, 54] pulmonary hypertension [22, 23] and the general population [55], and provides additional insight into the impact of IPEDs on the heart. We demonstrate lower reservoir and conduit LA strain in current users compared to both past and non-users of IPEDs, alongside lower reservoir and conduit RA strain in current users compared to non-users. Reduced LA reservoir strain is associated with increased stroke risk in both heart failure patients with sinus rhythm [54] and the general population [56, 57]. Similarly, LA reservoir and conduit strain predict cryptogenic stroke in both general and embolic stroke [55]. In hypertrophic cardiomyopathy patients, peak LA strain is predictive of 12-month outcome regarding death and/or hospitalisation [58]. Atrial compliance and stiffness govern both conduit and reservoir strain [59], thus, considering the differences in LA stiffness between current, past, and non-users of IPEDs in the current study, it is feasible to suggest that this likely played a role.

Low LA strain has been inversely correlated with degree of fibrosis through electromechanical mapping [60], contrast enhanced MRI [47] and histopathology [61, 62]. This suggestion is supported by multiple studies finding interstitial, perivascular and subendocardial fibrosis in IPED users [63–65]. Fibrosis in IPED users is suggested to be caused by multiple factors. Rapid myocardial hypertrophy can cause the myocardium to outgrow angiogenesis rates, resulting in necrosis and therefore fibrosis [66, 67]. In support, rat [68] and neuron-like cell [69] studies confirmed a role of apoptosis in IPED-related damage, causing fibrosis through necrosis [67]. Myocardial inflammation causes fibrosis through activation of profibrotic pathways [67] and, in mice, IPED administration caused increased myocardial expression of inflammatory cytokine interleukin 1-β and tumour necrosis factor-α showed more extensive expression [70] with a human autopsy study confirming the presence of inflammatory infiltrates in the myocardium of IPED users with sudden cardiac death [11]. Fibrosis acts as an arrhythmogenic substrate and may cause the increased rate of sudden cardiac death in IPED users [71] and therefore further studies to determine the nature of any involvement in the intrinsic reduction in atrial function is recommended.

Following diagnosis of IPED induced pathology, the primary treatment is immediate cessation of use [72]. Baggish et al. [13] found that following a period of IPED cessation, LV systolic function improved, with past users demonstrating similar LVEF to non-users, whereas diastolic function remained impaired, with decreased LV early relaxation velocity. The current study found no difference in LA and RA reservoir and conduit strain or LA volume and RA area between past and non-users of IPEDs. Although this cannot be definitively attributed to IPED cessation due to the cross-sectional nature of the study, it is supportive of past users showing a degree of functional and structural recovery following IPED cessation. A small case series (n = 3) found an improvement in LV diastolic function during the “off” period of an IPED cycle [73]. Additionally, due to the correlation between LA conduit strain, LA stiffness, RA peak strain and RA booster strain and IPED dose, drug cycling may transiently affect functional parameters. Therefore, larger longer duration longitudinal studies of greater sample size are required to clarify the impact of IPED cessation and drug cycle timing on atrial structure and function and ultimately cardiac risk.

The recovery of atrial structure and function could be attributed to multiple factors. Firstly, attenuation of IPED induced interstitial fibrosis may lead to improvements in intrinsic function and compliance. In a mice model of fibrotic interstitial cardiomyopathy due to ischemia, discontinuation of the ischemia protocol resulted in fibrosis reversal [74]. Similarly, in patients with LV hypertrophy and diastolic dysfunction, treatment with an angiotensin-converting enzyme inhibitor over 6-months attenuated fibrosis with an association between fibrosis reduction and improved diastolic function [75]. Studies are needed to directly measure fibrosis following IPED cessation to clarify this link. Secondly, differences in diastolic dysfunction between current users and past users, as found in the current study (A wave, E/A ratio and average E/E’) likely contributes to the reversal of pathological structural and functional remodelling following IPED cessation. In a study of heart failure patients (n = 107), bi-atrial pump function (emptying fraction) was significantly improved and LA size was reduced following cardiac resynchronisation therapy due to pressure and volume unloading effects from reverse ventricular remodelling [76]. Studies have found similar improvements in atrial structure and function due to decreased atrial loading following valve replacement [77] and in AF patients following ablation [78]. Notably, previous studies have also found a lower systolic blood pressure and improvement in diastolic function in past users compared to current users [79, 80] [81, 82]. As LA strain has been demonstrated as a surrogate of LV diastolic function [18], decreases in blood pressure and thus afterload with cessation of IPED use may also be responsible for the decrease in LA strain between past and current users. Further research is needed to investigate this in IPED users. Additionally, it is unclear whether reversal of remodelling results in decreased mortality risk and sudden cardiac death in IPED users [83] and the extent of reverse remodelling may be influenced by duration and dose of IPED use [13, 84]. It is plausible to suggest that the response to cessation will differ between individuals depending on the extent of disease [67].

### Limitations

There are some limitations to this study. Absolute IPED use and training volumes were self-reported and hence are potentially subject to bias. That aside participants were fully aware of the aims and nature of the study and were generally actively open regarding their substance use. Also, sensitivity and specificity of self-report in the detection of urinary anabolic steroids has previously been found to be high [85]. Nevertheless, the authenticity of IPEDs used is an ongoing issue.

Additionally, polypharmacy in all participants means that the effects of specific substances cannot be discerned. It is important to note that this is representative of IPED use and highlights the impact of the regime / cocktail of drug use in this population. Between group differences in atrial structural parameters normalised following allometric scaling, possibly suggestive of physiological remodelling, however, further studies could look at additional factors that could contribute to this within this unique population. Moreso, although there were no significant differences between training duration (years) or hours (per week), other factors such as training intensity or type may have caused differences in training load and cardiac demand between groups.

The cross-sectional nature of the study limits the applicability to long-term clinical implications and therefore any future studies should aim to address longitudinal changes, adverse outcomes, mortality and their association to specific IPED use.

### Conclusion

In conclusion, absolute LA volume and RA area are higher in current users of IPEDs than both past and non-users, however, when scaled allometrically for BSA these differences were removed and therefore these differences could be associated with an increased muscle mass. Atrial reservoir and conduit strain are reduced, and LA stiffness is higher in current users than past and non-users. There was no difference in LA and RA reservoir and conduit strain alongside LA volume and RA area between past and non-users, although this cannot be definitively attributed to IPED cessation due to the cross-sectional nature of the study. The outlined findings of potential pathological adaptation, alongside the recent rise in IPED use and high use within gym communities, highlights the clinical importance of the current study.

## Data Availability

The datasets generated and/or analysed during this study are not publicly available in order to protect privacy.
